# Horizontal and Vertical Distribution of Perfluoroalkyl Acids (PFAAs) in the Water Column of the Atlantic Ocean

**DOI:** 10.1021/acs.estlett.3c00119

**Published:** 2023-04-12

**Authors:** Eleni K. Savvidou, Bo Sha, Matthew E. Salter, Ian T. Cousins, Jana H. Johansson

**Affiliations:** † Department of Environmental Science, Stockholm University, 106 91 Stockholm, Sweden; ‡ Department of Thematic Studies − Environmental Change, Linköping University, 581 83 Linköping, Sweden; § Bolin Centre for Climate Research, Stockholm University, 106 91 Stockholm, Sweden

**Keywords:** PFOA, PFOS, seawater, depth profiles, hemisphere, Mediterranean Sea, gyre, English Channel

## Abstract

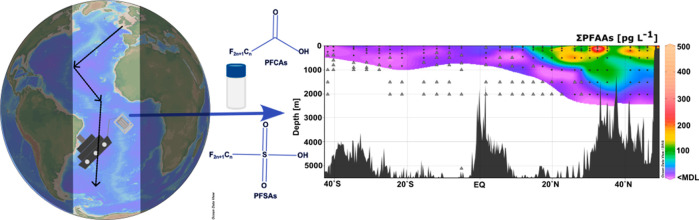

Perfluoroalkyl acids (PFAAs) are widely distributed in the oceans which are their largest global reservoir, but knowledge is limited about their vertical distribution and fate. This study measured the concentrations of PFAAs (perfluoroalkyl carboxylic acids (PFCAs) with 6 to 11 carbons and perfluoroalkanesulfonic acids (PFSAs) with 6 and 8 carbons) in the surface and deep ocean. Seawater depth profiles from the surface to a 5000 m depth at 28 sampling stations were collected in the Atlantic Ocean from ∼50° N to ∼50° S. The results demonstrated PFAA input from the Mediterranean Sea and the English Channel. Elevated PFAA concentrations were observed at the eastern edge of the Northern Atlantic Subtropical Gyre, suggesting that persistent contaminants may accumulate in ocean gyres. The median ΣPFAA surface concentration in the Northern Hemisphere (*n* = 17) was 105 pg L^–1^, while for the Southern Hemisphere (*n* = 11) it was 28 pg L^–1^. Generally, PFAA concentrations decreased with increasing distance to the coast and increasing depth. The C6–C9 PFCAs and C6 and C8 PFSAs dominated in surface waters, while longer-chain PFAAs (C10–C11 PFCAs) peaked at intermediate depths (500–1500 m). This profile may be explained by stronger sedimentation of longer-chain PFAAs, as they sorb more strongly to particulate organic matter.

## Introduction

Comparisons of the relative amounts of perfluoroalkyl acids (PFAAs) in multiple environmental reservoirs^1,2^ and global transport and fate modeling studies^3−10^ have determined that the global oceans are the main environmental reservoir for PFAAs. Understanding the spatial distribution in global oceans is key to projecting how environmental levels respond to changed emissions^9^ and to modeling ocean-to-atmosphere transport of PFAAs through sea spray aerosols (SSA).^11−13^ As there is currently no oceanic monitoring program for PFAAs, current knowledge is built on data generated from research cruises.^14−25^ Although numerous such studies have been carried out, the spatial distribution of data points in open oceans is insufficient for verifying high spatially resolved ocean transport models^9^ or for accurate prediction of atmospheric emission of PFAAs via SSA.^11,12^ A recent review of PFAAs in the global oceans revealed that the coastal areas of western Europe, China, Korea, and Japan account for most of the available concentration data.^26^ Additionally, the scientific studies that make up the body of data have been carried out by different research groups over the course of an almost 20-year period, during which time analytical methods have improved significantly.^26,27^

In lieu of high spatial resolution surface seawater measurements, vertical profiles along a cruise transect can provide valuable information on the oceanic circulation of PFAAs and thus help in interpolation between surface water data points. Previously, only a few such studies have been carried out.^15,23,25,28,29^ Due to a low number of data points and/or low detection frequencies, these have generally not been able to provide a high-resolution spatial distribution of oceanic PFAAs.^15,23^ Moreover, due to contradictory observations, there is a debate as to whether PFAAs act as chemical tracers for ocean circulation^15^ or sorb to particulate organic matter and sediment to deeper waters.^30−32^ This illustrates the need for a better understanding of the fate of PFAAs in the ocean. More extensive deep-water measurements are needed for a range of PFAA homologues to elucidate the processes that control their vertical transport. Therefore, this study aimed to (1) extensively investigate the spatial distribution (horizontally and vertically) of PFAAs (perfluoroalkyl carboxylic acids (PFCAs) and perfluoroalkanesulfonic acids (PFSAs)) in the Atlantic oceanic water column and, through thorough interpretation of the data, (2) clarify the ongoing debate concerning their fate and behavior in the oceanic environment.

## Materials and Methods

**Figure 1:**
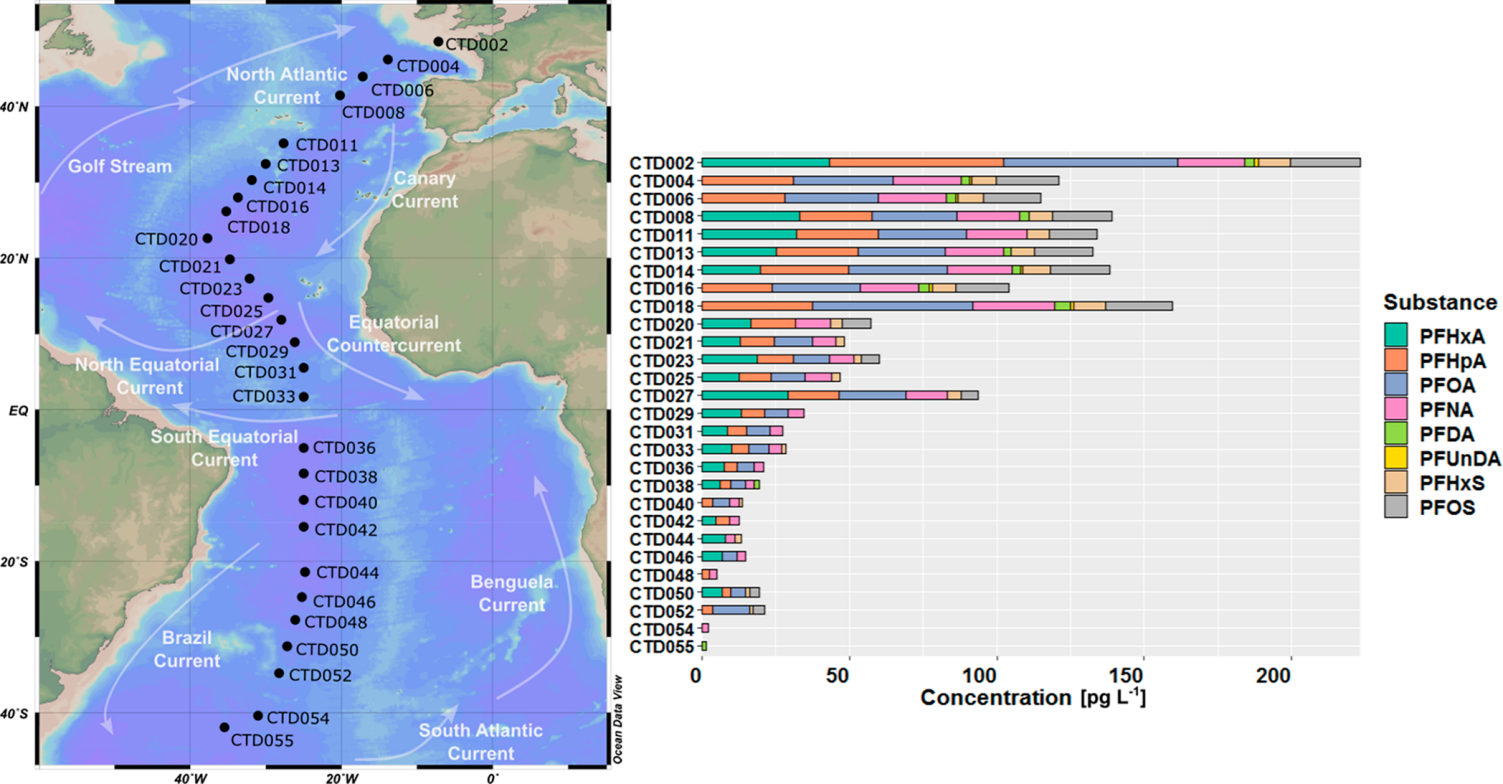
Map of the sampling stations along the transect and composition pattern of PFAAs (PFAAs below the detection limit are excluded) in the surface water samples (2 and 5 m) at the sampling stations.

Seawater samples were collected on the 29th Atlantic Meridional Transect (AMT29) cruise, from Southampton (UK) to Punta Arenas (Chile) in the time period from October to November 2019. Depth profile samples were obtained at 28 stations with a conductivity, temperature, and depth (CTD) rosette containing 24 × 20 L Ocean Test Equipment (OTE) Niskin bottles (Figure 1). At each station, samples were taken from seven varying depths ranging from the surface down to 5000 m (see Table S1 for exact depths). Unfiltered seawater was extracted on Oasis weak ion exchange (wax) cartridges, using a previously published solid phase extraction method (SPE),^33^ with minor modifications (see the Supporting Information (SI)). The samples (5 L) were loaded onto SPE cartridges on board using a multichannel peristaltic pump and then eluted later in the laboratory at Stockholm University. Field blanks were prepared by circulating 5 mL of Milli-Q through an SPE cartridge during the extraction of the samples (see SI). The analysis was performed on a Dionex Ultimate 3000 liquid chromatograph coupled to a Q Exactive HF Orbitrap (Thermo Scientific). The CTD samples (*n* = 196) were analyzed for 12 PFAAs (C6–C12). Due to contamination from reagents, all data points were blank subtracted for perfluorooctanoic acid (PFOA), while a separate batch of samples were blank subtracted for perfluorohexanoic acid (PFHxA). Additionally, a CTD cast (CTD034; 35° W, 26° N) had to be removed from the data set due to this reagent contamination, resulting in 28 representative sampling stations instead of originally 29 (see SI). A full description of the materials and methods as well as of the batches is provided in the SI.

## Results and Discussion

### Trends of PFAA Concentrations in the Surface of the Water Column

Of the 12 PFAAs targeted, eight were detected in surface samples (*n* = 28), collected at 2 or 5 m (Figure 1, Table S1). Perfluorononanoic acid (PFNA) had the highest detection frequency in surface water (*n* = 28) with 89%, followed by perfluoroheptanoic acid (PFHpA) (86%) and PFOA (78%). PFHxA, perfluorohexanesulfonate (PFHxS), perfluorooctanesulfonate (PFOS), perfluorodecanoic acid (PFDA), and perfluoroundecanoic acid (PFUnDA) had detection frequencies of 64%, 68%, 50%, 39%, and 21%, respectively. Perfluorobutanoic acid (PFBA), perfluoropentanoic acid (PFPeA), perfluorododecanoic acid (PFDoDA), and perfluorobutanesulfonic acid (PFBS) were omitted from the study due to poor recoveries.

PFAA concentrations decreased from the coast to the open ocean (Figure 1). This has been observed in previous studies and is known as the dilution effect.^16,19,20^ The highest ΣPFAA surface concentration (224 pg L^–1^) was measured at 45° N (CTD002, Figure 1) in this study. The higher concentrations at CTD002 compared to the rest of the sampling sites were probably due to the greater proximity to the coast. A bioaccumulation study on shellfish from this area also indicated extensive contamination with PFAAs, especially PFOS and long-chain PFCAs.^34^ The findings of high PFAA contamination in the English Channel from this and previous studies indicate that the English Channel is a potentially important source of PFAAs in the open Atlantic Ocean.

**Figure 2:**
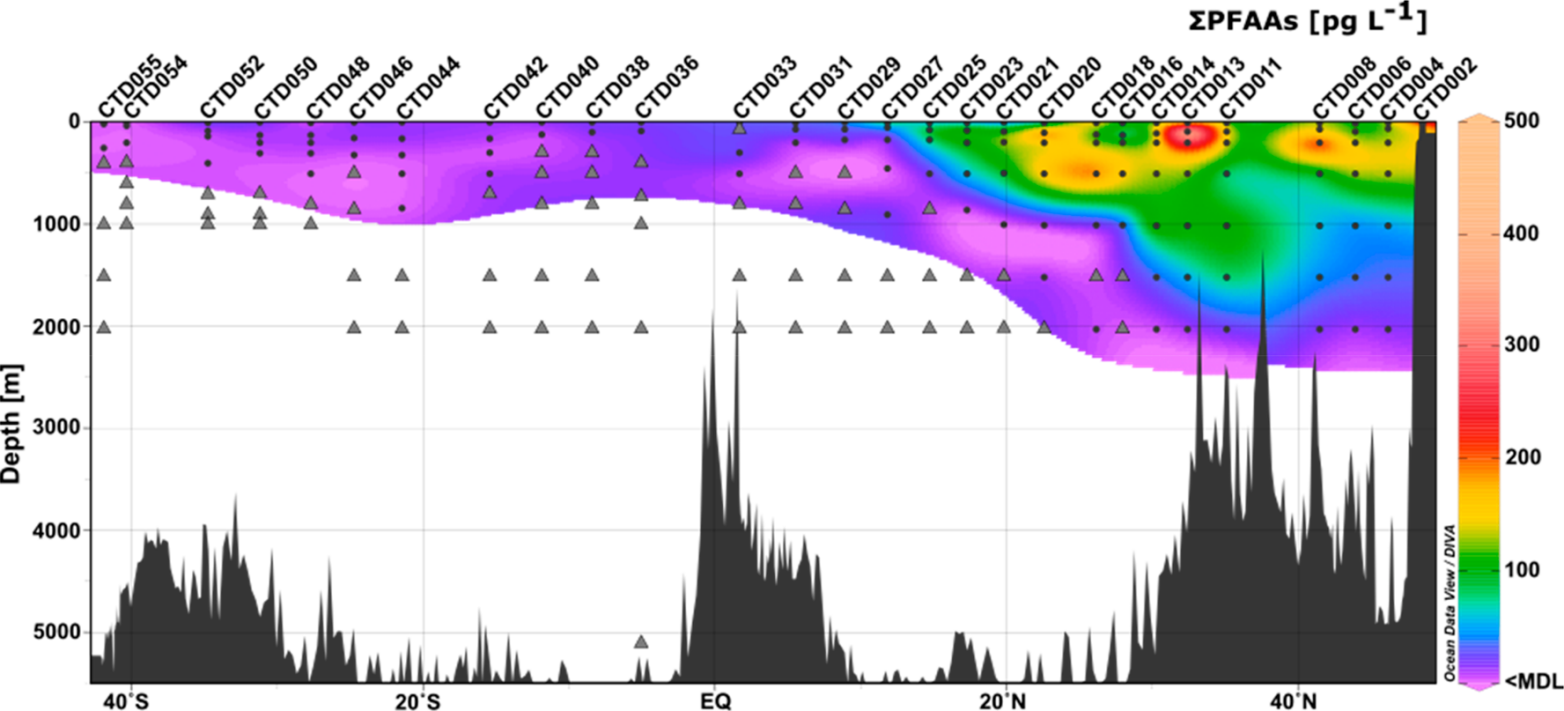
Spatial distribution of the sum of PFAAs in the northern and southern hemisphere of the Atlantic Ocean. Black dots represent sample points where ΣPFAAs could be detected; gray triangles represent nondetects.

The detection frequency was higher for all substances in the Northern Hemisphere (NH) compared to the Southern Hemisphere (SH). The median concentrations of ΣPFAAs in surface water were 105 pg L^–1^ (*n* = 17) and 28 pg L^–1^ (*n* = 11) in the NH and SH, respectively (Figure 2). The concentrations were shown to be statistically significantly lower in the SH compared to the NH for all PFAAs (Wilcoxon test, *p* < 0.05) (note that there were fewer sampling stations in the SH). Lower open ocean levels are expected in the SH, since most of the global manufacture and industrial use of PFAAs has occurred in the US, Europe, Japan, and China.^7^ This is also observed in other studies where concentrations of PFAAs decreased to levels below detection limits for most cruises that followed transects from the Northern to the Southern Atlantic.^16,18,19^ These previous studies were mostly limited to coastal samples, and therefore, it is difficult to compare them with open ocean data from the present study. This highlights the difficulty in interpolating the data currently available in the published literature to obtain estimates of seawater concentrations representative of larger open ocean regions. Contrary to other studies, González-Gaya et al. (2014) reported a higher median ΣPFAS concentration in the surface water in the SH (1440 pg L^–1^) compared to the NH (516 pg L^–1^) of the Atlantic Ocean.^20^ Differences in concentrations can be due to different sampling locations and the size of the sample set.

In our study, PFOS was detected throughout the English Channel and the North Atlantic southward to 10° N (CTD027, Figure 1). However, in the SH, the detection frequency for PFOS was only 18%, and this substance was observed in only two samples between 30° S and 40° S (CTD050 and CTD052, Figure 1). Although it was predicted that surface removal of PFOS would lead to higher concentrations in deeper water layers over time,^9^ this study does not confirm such a trend. Other homologues such as PFNA (73%) and PFHpA (64%) had higher detection frequencies in the SH (note though that their MDLs were somewhat lower than that of PFOS, see SI). In addition to absolute concentrations, trends in homologue patterns can also be influenced by the choice of sampling location. As coastal areas are closer to emission sources, coastal waters may contain relatively higher concentrations of homologues that sorb to the particulate organic matter present in the water column. This is a possible explanation for the discrepancy between our results and previous studies reporting PFOS as the dominant compound on the surface of the SH.^17,19,20^ High concentrations of PFOS have previously been measured off the Brazilian coast^19,20,33^ and are suspected to be related to the widely used pesticide Sulfluramid in South America, which degrades in the environment to form PFOS and other PFAS.

The source of PFOS in the South Atlantic Ocean observed in our data set between 30° S and 40° S (CTD050 and CTD052, Figure 1) could be the outflow of the Rio de la Plata, which is a heavily polluted river.^35^ In particular, pulp mills, which are common in Uruguay, could be a potential source of PFOS and other PFAS.^36^ In a previous study by Langberg et al. (2021), the potential role of a pulp mill in Norway in PFOS contamination of a lake was highlighted.^37^ However, the Rio de la Plata catchment area is highly populated and industrialized, making it difficult to pinpoint a specific source that could be responsible for the elevated concentrations of PFOS in these two CTD casts. For example, Sulfluramid could also be a source of PFOS in the Rio de la Plata, as it is imported from Brazil and used in Argentina and Uruguay.^38^

A subsurface sample (95 m) at approximately 30° N (CTD013, Figure 1) showed elevated PFAA concentrations. The sampling point is located in the eastern edge- or so-called Azores Front^39^ of the Northern Atlantic Subtropical Gyre. This region receives input from North America and Europe^40−42^ and is known for its plastic pollution,^43^ as plastic gets trapped in the gyre and forms a patch (often referred to as the North Atlantic Garbage Patch).^41^ Subsequently, plastic particles can sink from the surface to deep water.^44^ Previous studies have indicated that plastic debris can act as carriers of organic contaminants such as polychlorinated biphenyls (PCBs), dichlorodiphenyltrichloroethane (DDT), dichlorodiphenyldichloroethylene (DDE), nonylphenols (NPs), and polycyclic aromatic hydrocarbons (PAHs).^45,46^ Furthermore, studies have shown the sorption potential of PFOS and perfluorooctanesulfonamide (FOSA) in microplastics.^47,48^ Our finding could be the first indication that chemicals can potentially get trapped in this gyre via plastic debris.

### Trends of PFAA Concentrations with Depth and Their Vertical Distribution

In the following section, the concentrations for the depth samples taken from intermediate depths (500–1500 m; *n* = 80) and deep waters (1500 m to bottom; *n* = 24) are presented and discussed. Apart from PFDoDA, all target compounds could be detected at depth (*n* = 104). The detection frequency of ΣPFAAs (one PFAA or more in a sample) for the intermediate depths was 48%, while for deep water it was only 29%. Including all depths, the detection frequency was highest for PFHpA (34%), followed by PFUnDA (32%). The lowest detection frequency was observed for PFOA (17%). Compared to surface samples, the detection frequency for PFUnDA increased with depth, while for PFDA it dropped. However, generally with increasing depth, a decrease in concentration was observed for C6–C9 PFCAs as well as C6 and C8 PFSAs, while for the longer-chain PFAAs (C10–C11 PFCAs) concentrations increased with increasing water depth.

Between 40° N and 30° N at 500–1500 m depth in the NH, concentrations were elevated relative to surrounding areas. In combination with oceanographic data (Figures S1 and S2) obtained from the CTD measurements, it appears that this elevated concentration area (CTD011–014) was Mediterranean Outflow Water (MOW). This indicates a major input of PFAAs from the Mediterranean Sea into the Atlantic Ocean. Previously, studies have concluded that the Atlantic Ocean is a source of contamination to the Mediterranean Sea^49^ or that there is no significant flow of PFAAs between the water bodies.^28^ These conclusions were based on comparisons of surface water measurements made in different studies in the Mediterranean Sea^28,49^ and the Atlantic Ocean.^17,19,20^ However, since outward transport of Mediterranean seawater into the Atlantic Ocean occurs at intermediate depth (500–1500 m) as well as at the surface,^50^ comparisons of surface water measurements alone are not suitable to determine the flux of PFAAs between the two water bodies.

**Figure 3:**
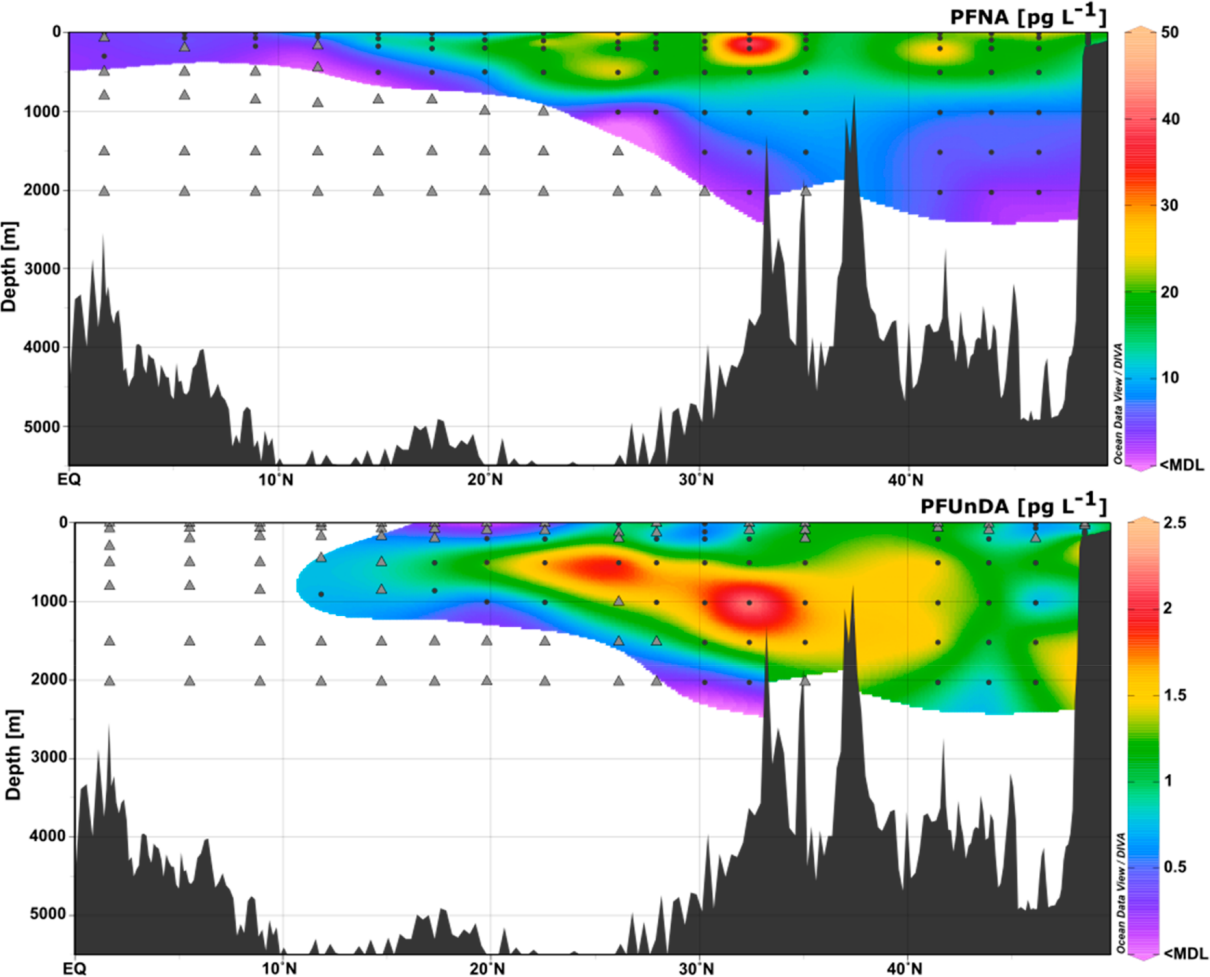
Distribution of PFNA in the vertical water column in the Northern Hemisphere. Black dots represent the sampling stations; gray triangles are nondetects (<MDL).

Due to the low detection frequencies at depth in the SH, our discussion on vertical profiles focuses on the NH between 50° N and the equator. In Figure 3, the vertical profiles of PFNA and PFUnDA are depicted as an example (for other homologues, see SI Figures S3–S9). Generally, PFAA concentrations at all depths decreased toward the equator. Exceptions from this trend were observed around 500 m at 25° N and 1500 m at 35° N (MOW) where concentrations of PFHxA, PFOA, PFHxS, and PFOS were higher than in surface waters. PFHxA, PFHpA, PFOA, PFNA, and PFHxS showed similar distribution patterns. Concentrations of C6–C9 decreased with depth at latitudes from 50° N to 40° N. From around 28° N toward the equator, concentrations at depths below 500 m were mainly below the MDL. While PFAAs with chain lengths between C6 and C9 were mostly concentrated on the surface (0–500 m), the distribution pattern of PFUnDA looked different. PFUnDA had nondetects at the surface throughout most of the sampling stations. Higher concentrations of PFUnDA were observed from 50 °N to 20° N in intermediate waters (800–2000 m). This could be explained by the higher organic carbon–water partition coefficient (*K*_OC_) of PFUnDA in relation to the other homologues.^51^ The propensity to sorb to particulate organic matter can allow PFUnDA to sediment out of surface waters. PFDA showed an irregular distribution pattern, having concentration hotspots and nondetects at a few sampling points in the surface, but also at 2000 m depth at around 30° N (CTD013). In the literature, the biogeochemical pump has been discussed as a mechanism for the surface removal of PFAA.^22^ The increase in concentration around 30° N (CTD013, Figure 1) might indicate that PFUnDA sorbed to particulate matter or microplastic sinks from the Gyre/Azores front. This could also be true for PFDA (Figure S6). Our findings suggest that downward particle transport occurs for PFAAs with chain lengths that contain 10 or more carbon atoms. As such, these are inappropriate chemical tracers for ocean circulation. However, a complication is that PFCAs and PFSAs have multiple direct and indirect sources,^7^ making interpretation of contamination patterns in the ocean challenging.

In conclusion, PFAA concentrations were higher in the NH compared to the SH, and there was a general decrease in PFAA concentrations with increasing distance to the coast and increasing depth. Homologues with a higher adsorption affinity tended to sorb to particulate matter and sink down the water column. Furthermore, vertical profiles enabled the Mediterranean to be identified as a likely source of PFAAs to the Atlantic, along with the English Channel. Finally, PFAAs were considered to be unsuitable ocean circulation tracers due to their complex origin of sources and partitioning behavior within the water column, i.e., their interaction with sinking particulate organic matter.

## Supporting Information Available

Additional information on materials and methods including additional tables and graphs (PDF)
All CTD data and concentrations (XLSX)
