# Supplementary material for: Horizontal and Vertical Distribution of Perfluoroalkyl Acids (PFAAs) in the Water Column of the Atlantic Ocean
Source: Environ Sci Technol Lett. 2023 Apr 12;10(5):418–24. doi: 10.1021/acs.estlett.3c00119 (PMC10173459; doi:10.1021/acs.estlett.3c00119)
Supplement: Supplementary file 1 — ez3c00119_si_001.pdf [file ez3c00119_si_001.pdf]

Supporting Information

Horizontal and vertical distribution of perfluoroalkyl acids (PFAAs) in the water column of the Atlantic Ocean

Eleni K. Savvidou[1*], Bo Sha[1], Matthew E. Salter[1,3], Ian T. Cousins[1] and Jana H. Johansson[2]

*Corresponding Author: eleni.savvidou@aces.su.se

[1]Department of Environmental Science, Stockholm University, Stockholm, Sweden

[2]Department of Thematic Studies – Environmental Change, Linköping University, Linköping, Sweden

[3]Bolin Centre for Climate Research, Stockholm, Sweden

Methods and Materials

Oceanographic setting/water masses

The data on salinity and temperature obtained from every CTD cast could be used to reconstruct and identify the different water masses that were sampled along the cruise. The water masses were characterized based on the properties described in Table S2 and are presented in figure S1. In the Northern Hemisphere, the surface water (0 until 500 m) is mostly dominated by North Atlantic Central Water (NACW), or more specifically East North Atlantic Central Water (ENACW). It starts at around 50˚N, and further expands down until 10˚N. From 40˚N until 10˚N very high salinities (37-38 g kg^{-1}) and temperatures were observed, which is characteristic for the so called Northern Subtropical Gyre (NSTG). From 50˚N until 30˚N latitude, the Mediterranean Outflow Water (MOW) was visible in intermediate depths. It is more saline, and therefore denser than the ENACW, so that when the MOW flows out the Strait of Gibraltar, it sinks down, and occupies intermediate depths (here 500-1200 m) .[1] Close to the equator at around 10˚N, water from the Southern Hemisphere, i.e. South Atlantic Central Water (SACW) was predominately sampled. The water mass with a relatively low salinity (Figure S1, indicated in green, 35.0-35.5 g kg^{-1}) is the North Atlantic Deep Water (NADW) and expands until 30˚S. Crossing the equator down to 30˚S, the warm and saline water characterized as the Southern Subtropical Gyre (SSTG) which is composed of SACW was sampled. In the intermediate depths of the South Atlantic of 500 down to 2000 m the Antarctic Intermediate Water (AAIW) was occurring and is reaching latitudes up to 10˚N.

Chemicals

Chemicals used were acetonitrile, ammonium acetate (ProAnalysis), formic acid (98%, Reag. PhEur), methanol (MeOH) which were all purchased from Merck. 25% ammonium hydroxide solution was obtained from Sigma-Aldrich. The MilliQ water was obtained from a Millipore water purification system. Furthermore, native and mass-labelled PFCA and PFSA standards were purchased from Wellington (Table S3).

The target analytes of this study were perfluorobutanoic acid (PFBA), perfluoropentanoic acid (PFPeA), perfluorohexanoic acid (PFHxA), perfluoroheptanoic acid (PFHpA), perfluorooctanoic acid (PFOA; branched and linear), perfluorononanoic acid (PFNA), perfluorodecanoic acid (PFDA), perfluoroundecanoic acid (PFUnDA), perfluorododecanoic acid (PFDoDA), perfluorobutanesulfonic acid (PFBS), perfluorohexanesulfonic acid (PFHxS) and perfluorooctanesulfonic acid (PFOS; branched and linear).

Instrumental analysis

The mobile phases used for the chromatographic part were A) water:acetonitrile (90:10, 2 mM ammonium acetate), and B) acetonitrile:water (99:1, 2 mM ammonium acetate). The gradient program for the mobile phases is shown in Table S4. A PFAS isolator pre-column was connected prior to the loop in order to eliminate potential contamination from the mobile phases and the system. The separation of the analytes was performed on an Acquity URLC® BEH C18 column (1.7 µm, 2.1x50 mm, Waters®). The oven temperature for the column was set to 50 ℃. The flow rate was set to 0.4 mL min^{-1} and the injection volume was 25 µL. The

instrument was operated in negative ionization mode, using electrospray ionization (ESI). Further instrumental settings are listed in Tables S4 and S5.

Sample treatment

A previously published solid phase extraction (SPE) method by Giljam et al. (2016)[2], which is based on weak anion exchange, was modified and used for the extraction of the samples. Prior to the cruise, the cartridges (Oasis®Wax, 6 cm^3, 500mg, 30μm) were washed with 4.5 mL of 0.3% ammonium hydroxide in MeOH, and then activated with 4.5 ml of 0.1 M formic acid in MilliQ water. Then the cartridges were wrapped in aluminum foil, sealed in zip bags and stored at 4 ℃ before use. The extraction of the samples was performed onboard. For this, 5 L samples were collected into polyethylene carboys, and then directly pumped through the cartridges with a multi-channel peristaltic pump at a flow rate of about 5-7 drops per second. After that, they were spiked with 25 μL internal standard (20 pg $μL^{-1}$). The samples were stored at –20 ℃ until further treatment and analysis. The samples were further processed in the laboratory at Stockholm University. The cartridges were washed with 5 mL of 20% MeOH:80% MilliQ water and 2 mL of 0.3 % ammonium hydroxide in MilliQ water. Vacuum condition was used to initiate the flow of the solutions if needed. Afterwards, the cartridges were eluted with 6 mL of 0.3% ammonium hydroxide in MeOH. The samples were evaporated to <100 μL under gentle nitrogen flow. Afterwards, 150 μL MeOH, and a few drops of water buffer (4 mM ammonium acetate) were added to a final volume of approximately 300 μL. The extracts were swirled to fully mix, and ultrasonicated for 15 minutes. Then they were filtered using centrifugal filters (0.2 μm, nylon filters, VWR) at 10 000 rpm for 10 minutes. After that, they were transferred to 300 μL vials and 20 μL of recovery standard (20 pg $μL^{-1}$) was added.

Quality assurance

For the spike-recovery and breakthrough test, five 5 L canisters were washed twice with tap water and three times with MilliQ water. The five canisters were filled with 5 L MilliQ water, and 175 g of NaCl were added to obtain a salinity of 35 g L^{-1}. Three of the canisters were spiked with 1200 ng each of TFA and PFPrA and 500 pg of a mixture of C4-C12 PFCAs, and C4, C6 and C8 PFSAs. They were left overnight for equilibration. Cartridges were prepared as described in the section above. To load the sample onto the cartridge, a multi-channel peristaltic pump was used with a flow rate of approximately 2-3 drops per second. Two cartridges were connected in series to examine if there was any breakthrough of the target analytes. After the loading, 1200 ng of an internal standard of TFA and 500 pg of the native long-chain substances were added directly to every cartridge. Then they were washed with 5 mL of 0.1% formic acid in MeOH, and 2 mL of 0.3 % ammonium hydroxide in MilliQ water. Afterwards, the samples were eluted with 5 mL of 0.3% ammonium hydroxide in MeOH, and then evaporated under gentle nitrogen flow. They were reconstituted with 130 μL MeOH and 150 μL water buffer, followed by filtration to remove solid particles. Then, the extracts were transferred to vials and 20 μL recovery standard (20 pg $μL^{-1}$) was added.

For the PFCAs and PFSAs, with carbon numbers from C5-C12 and C4-C8, respectively, spike recovery tests had been conducted prior to the cruise, and showed satisfactory results with

recoveries ranging from 52-106 %. The results for these compounds are summarized in Table S6.

The recoveries of the mass labelled internal standards were estimated to evaluate the performance of the extraction and analysis methods (Table S7) used. The concentrations of the internal standards were calculated using the recovery standard ($^{13}C_8$-PFOA and $^{13}C_8$-PFOS). The recovery was obtained by dividing the measured concentration by the expected (spiked) concentration. PFCAs with carbon chain-lengths from C8-C9 had median recoveries ranging from 83-85%, which indicates that the SPE method was suitable for these homologues. A similar trend was observable for the PFSA homologues MPFHxS and MPFOS with 77% and 79% recovery, respectively. C6 and C7 had low median recoveries with 37% and 46%, respectively. This might be due to matrix suppression, i.e. the signal of the analytes is suppressed due to interference of other substances during ionization. In the case of the longer-chain substances (C10-C12) matrix enhancement might be the reason for the high recovery ranges that were observed (maximum recoveries ranged from 275-372%), although the medians were in an acceptable range.

Field blanks were obtained by letting 5 mL of MilliQ circulate through a cartridge while the samples of the respective CTD cast were loaded onto SPE cartridges. Laboratory blanks were prepared when extracting the samples after the cruise by spiking 25 µL internal standard (20 pg $µL^{-1}$) directly onto cartridges without the addition of MilliQ water. They were made using cartridges which had been conditioned prior to the cruise.

This preparation procedure was carried out in four batches named A-D (Table S8). Based on the laboratory and field blanks that showed levels of the target analytes, the method detection limit (MDL) was calculated by using the mean plus three times the standard deviation. To determine the method quantification limit (MQL), the mean plus ten times the standard deviation was used. Where there was no substance in the blanks of a specific batch detectable, the MDL and MQL of another batch was chosen. The respective MDL and MQL for each target substance in the four different batches are summarized in Table S9.

Contamination with PFOA and PFHxA

Elevated PFOA concentrations were observed in tests with CTD034, and in the laboratory blanks when the original method by Giljam et al. (2016) was used. Investigations showed that the PFOA contamination came from the formic acid, which was used to prepare and to wash the cartridges. The findings implied that the contamination was mainly introduced in the preparation of the cartridges before the cruise. The PFOA levels in the contaminated formic acid were around 1 pg $µL^{-1}$. Similar levels of PFOA were also found in a newly purchased bottle of formic acid. Therefore, to avoid introducing further contamination in the washing steps, it was tested to substitute the original solution of 0.1 M formic acid in 20% MeOH. Instead, a mixture of 20% MeOH: 80% MilliQ water was used as washing solution, and showed acceptable recoveries of the internal standards and blank levels in tests. Further, CTD034 was removed from the data set because of the contamination.

The cartridges had been prepared in three batches, so it was decided to perform a blank correction based on the blank level for each cartridge batch. The blank levels were estimated based on the laboratory blanks that were prepared from spare cartridges of batch 1 and 2.

There were no cartridges left from batch 3 to proceed the same way. In that case, the samples from the deepest point (2000 m) of batch 3 were used to estimate the blank levels since they showed relatively consistent PFOA concentrations among the batch. The blanks of batch 1 and 2 and their respective samples from the deep ocean showed similar levels, further supporting the strategy applied on batch 3. The mean of the blanks for every batch was calculated, and then subtracted from every sample. In some cases, blank subtraction lead to negative values which were consequently considered as below the detection limit.

As the mean of the PFOA blanks was used to perform blank subtraction, the MDL was set as three times and the MQL as ten times the standard deviation of the blanks. As there were no blanks for batch 3 available, the MDL and MQL of batch 1 was chosen as the PFOA levels in the deep-water samples of batch 3 were in the same range as in batch 1. The results for PFOA are presented in Table S10.

Elution batch B showed elevated concentrations of PFHxA in the blanks, probably introduced during the washing and elution steps in the laboratory.

A blank correction was applied for PFHxA concerning the samples of elution batch B with the exception that both laboratory and field blanks were used to estimate the mean blank level.

In order to estimate the MDL and MQL for PFHXA in elution batch B, the same method as for PFOA was used.

Other PFAAs were not affected by contamination during the preparation or elution of the cartridges as they showed consistent blank levels throughout analysis. Thus, blank subtraction was not performed for PFAAs other than PFOA and PFHxA.

Variation in MDLs and MQLs

From the described calculation procedure above, the resulting MDLs and MQLs are batch specific which leads to a variation in values between batches. However, for most of the analytes the MDLs were fairly consistent between the different batches. A maximum discrepancy was observed for PFOS, as it had an MDL three times higher in Batch B (7.6 pg L^{-1}) compared to Batch C (2.5 pg L^{-1}). All samples from the same CTD cast were extracted within the same batch, but the individual CTD casts were randomized into different batches. As a result, the MDLs and MQLs are uniform vertically, whereas the vary randomly along the transect. When comparing MDLs (Table S9) and measured data (Figure S9), it shows that the batch-dependent MDLs have not introduced a trend or breaking point in detection frequency in the dataset, ultimately. The higher MDL batch corresponds to datapoints of the NH that were well above the MDL, and to non-detected datapoints of the SH that were surrounded by other non-detected datapoints.

Data treatment

The short-chain homologues PFBA, PFPeA and PFBS were removed from the dataset (including samples from the deep) due to poor recoveries and low-quality chromatograms.

In some deep water samples from the Southern Hemisphere (CTDs 029, 036, and 040), a few scattered values slightly above the method detection limit (MDL) were observed for PFHxA, PFHpA, PFOA, PFDA, PFDoDa and PFOS. However, as these values did not display any discernible trend and were surrounded by non-detects both vertically and horizontally, they were deemed as non-detects and subsequently excluded from the dataset.

For calculations of medians and the sum of PFAAs, the non-detects were included as half the respective MDL.

Data analysis

The targeted compounds were quantified with the software TraceFinder 4.1 based on their respective labelled internal standards (Table S3). The calibration was based on a linear regression, going through the origin and a weigh of 1/X. To examine the concentrations of the substances in context with the available oceanic data, the software Ocean Data View 5.3.0 was used. The input data consisted of the oceanic information that was obtained through the CTD measurements, including parameters such as temperature and salinity. In order to transform this collected information in commonly used oceanographic parameters (conservative temperature, absolute salinity), the tool TEOS-10 (Thermodynamic Equation of Seawater – 2010) was used. Additionally, the respective detection and quantification limits for each substance were added, and the individual samples were marked with quality flags (1: >MQL, Q: >MDL, 6: <MDL, 4: bad data, i.e. recoveries <10%, 9: not found in the chromatogram, 5: negative values, i.e. after blank correction) to have a better overview of the plotted data points.

Table S1. *CTD sample list from the AMT29 cruise with the respective dates, depths and coordinates.*

CTD No.	Date	Depth	Longitude	Latitude
CTD002	20191016	140m	-7.20367	48.52183
CTD002	20191016	120m	-7.20367	48.52183
CTD002	20191016	100m	-7.20367	48.52183
CTD002	20191016	70m	-7.20367	48.52183
CTD002	20191016	35m	-7.20367	48.52183
CTD002	20191016	5m	-7.20367	48.52183
CTD002	20191016	2m	-7.20367	48.52183
CTD004	20191018	2000m	-13.88232	46.1655
CTD004	20191018	1500m	-13.88232	46.1655
CTD004	20191018	1000m	-13.88232	46.1655

CTD004	20191018	500m	-13.88232	46.1655
CTD004	20191018	200m	-13.88232	46.1655
CTD004	20191018	70m	-13.88232	46.1655
CTD004	20191018	5m	-13.88232	46.1655
CTD006	20191019	2000m	-17.14783	43.91417
CTD006	20191019	1500m	-17.14783	43.91417
CTD006	20191019	1000m	-17.14783	43.91417
CTD006	20191019	500m	-17.14783	43.91417
CTD006	20191019	200m	-17.14783	43.91417
CTD006	20191019	90m	-17.14783	43.91417
CTD006	20191019	5m	-17.14783	43.91417
CTD008	20191020	2000m	-20.18851	41.4655
CTD008	20191020	1500m	-20.18851	41.4655
CTD008	20191020	1000m	-20.18851	41.4655
CTD008	20191020	500m	-20.18851	41.4655
CTD008	20191020	200m	-20.18851	41.4655
CTD008	20191020	70m	-20.18851	41.4655
CTD008	20191020	5m	-20.18851	41.4655
CTD011	20191023	2000m	-27.62466	35.09133
CTD011	20191023	1500m	-27.62466	35.09133
CTD011	20191023	1000m	-27.62466	35.09133
CTD011	20191023	500m	-27.62466	35.09133
CTD011	20191023	200m	-27.62466	35.09133
CTD011	20191023	90m	-27.62466	35.09133
CTD011	20191023	5m	-27.62466	35.09133
CTD013	20191024	2000m	-30.02383	32.402
CTD013	20191024	1500m	-30.02383	32.402
CTD013	20191024	1000m	-30.02383	32.402
CTD013	20191024	500m	-30.02383	32.402
CTD013	20191024	200m	-30.02383	32.402
CTD013	20191024	95m	-30.02383	32.402
CTD013	20191024	5m	-30.02383	32.402
CTD014	20191025	2000m	-31.86084	30.26767

CTD014	20191025	1500m	-31.86084	30.26767
CTD014	20191025	1000m	-31.86084	30.26767
CTD014	20191025	500m	-31.86084	30.26767
CTD014	20191025	200m	-31.86084	30.26767
CTD014	20191025	115m	-31.86084	30.26767
CTD014	20191025	5m	-31.86084	30.26767
CTD016	20191026	2000m	-33.68466	27.95417
CTD016	20191026	1500m	-33.68466	27.95417
CTD016	20191026	1000m	-33.68466	27.95417
CTD016	20191026	500m	-33.68466	27.95417
CTD016	20191026	200m	-33.68466	27.95417
CTD016	20191026	120m	-33.68466	27.95417
CTD016	20191026	5m	-33.68466	27.95417
CTD018	20191027	2000m	-35.19482	26.13983
CTD018	20191027	1500m	-35.19482	26.13983
CTD018	20191027	1000m	-35.19482	26.13983
CTD018	20191027	500m	-35.19482	26.13983
CTD018	20191027	200m	-35.19482	26.13983
CTD018	20191027	120m	-35.19482	26.13983
CTD018	20191027	5m	-35.19482	26.13983
CTD020	20191028	2000m	-37.69934	22.58767
CTD020	20191028	1500m	-37.69934	22.58767
CTD020	20191028	1000m	-37.69934	22.58767
CTD020	20191028	500m	-37.69934	22.58767
CTD020	20191028	200m	-37.69934	22.58767
CTD020	20191028	105m	-37.69934	22.58767
CTD020	20191028	5m	-37.69934	22.58767
CTD021	20191029	2000m	-34.77768	19.79867
CTD021	20191029	1500m	-34.77768	19.79867
CTD021	20191029	1000m	-34.77768	19.79867
CTD021	20191029	500m	-34.77768	19.79867
CTD021	20191029	200m	-34.77768	19.79867
CTD021	20191029	95m	-34.77768	19.79867

CTD021	20191029	5m	-34.77768	19.79867
CTD023	20191030	2000m	-32.15134	17.279
CTD023	20191030	1500m	-32.15134	17.279
CTD023	20191030	850m	-32.15134	17.279
CTD023	20191030	500m	-32.15134	17.279
CTD023	20191030	200m	-32.15134	17.279
CTD023	20191030	80m	-32.15134	17.279
CTD023	20191030	5m	-32.15134	17.279
CTD025	20191031	2000m	-29.68433	14.73833
CTD025	20191031	1500m	-29.68433	14.73833
CTD025	20191031	850m	-29.68433	14.73833
CTD025	20191031	500m	-29.68433	14.73833
CTD025	20191031	170m	-29.68433	14.73833
CTD025	20191031	72m	-29.68433	14.73833
CTD025	20191031	5m	-29.68433	14.73833
CTD027	20191101	2000m	-27.96017	11.844
CTD027	20191101	1500m	-27.96017	11.844
CTD027	20191101	900m	-27.96017	11.844
CTD027	20191101	450m	-27.96017	11.844
CTD027	20191101	170m	-27.96017	11.844
CTD027	20191101	50m	-27.96017	11.844
CTD027	20191101	5m	-27.96017	11.844
CTD029	20191102	2000m	-26.19934	8.89167
CTD029	20191102	1500m	-26.19934	8.89167
CTD029	20191102	850m	-26.19934	8.89167
CTD029	20191102	500m	-26.19934	8.89167
CTD029	20191102	170m	-26.19934	8.89167
CTD029	20191102	70m	-26.19934	8.89167
CTD029	20191102	2m	-26.19934	8.89167
CTD031	20191103	2000m	-25.00168	5.52183
CTD031	20191103	1500m	-25.00168	5.52183
CTD031	20191103	800m	-25.00168	5.52183
CTD031	20191103	500m	-25.00168	5.52183

CTD031	20191103	200m	-25.00168	5.52183
CTD031	20191103	72m	-25.00168	5.52183
CTD031	20191103	2m	-25.00168	5.52183
CTD033	20191104	2000m	-25.00882	1.67667
CTD033	20191104	1500m	-25.00882	1.67667
CTD033	20191104	800m	-25.00882	1.67667
CTD033	20191104	500m	-25.00882	1.67667
CTD033	20191104	300m	-25.00882	1.67667
CTD033	20191104	76m	-25.00882	1.67667
CTD033	20191104	2m	-25.00882	1.67667
CTD034	20191105	2000m	-24.99966	-0.58117
CTD034	20191105	1500m	-24.99966	-0.58117
CTD034	20191105	750m	-24.99966	-0.58117
CTD034	20191105	500m	-24.99966	-0.58117
CTD034	20191105	260m	-24.99966	-0.58117
CTD034	20191105	75m	-24.99966	-0.58117
CTD034	20191105	2m	-24.99966	-0.58117
CTD036	20191106	5000m	-25.0015	-5.04367
CTD036	20191106	2000m	-25.0015	-5.04367
CTD036	20191106	1000m	-25.0015	-5.04367
CTD036	20191106	730m	-25.0015	-5.04367
CTD036	20191106	400m	-25.0015	-5.04367
CTD036	20191106	85m	-25.0015	-5.04367
CTD036	20191106	2m	-25.0015	-5.04367
CTD038	20191107	2000m	-24.9985	-8.406
CTD038	20191107	1500m	-24.9985	-8.406
CTD038	20191107	800m	-24.9985	-8.406
CTD038	20191107	500m	-24.9985	-8.406
CTD038	20191107	300m	-24.9985	-8.406
CTD038	20191107	100m	-24.9985	-8.406
CTD038	20191107	2m	-24.9985	-8.406
CTD040	20191108	2000m	-25.00766	-11.882
CTD040	20191108	1500m	-25.00766	-11.882

CTD040	20191108	800m	-25.00766	-11.882
CTD040	20191108	500m	-25.00766	-11.882
CTD040	20191108	300m	-25.00766	-11.882
CTD040	20191108	120m	-25.00766	-11.882
CTD040	20191108	2m	-25.00766	-11.882
CTD042	20191109	2000m	-25.00333	-15.4425
CTD042	20191109	1500m	-25.00333	-15.4425
CTD042	20191109	700m	-25.00333	-15.4425
CTD042	20191109	500m	-25.00333	-15.4425
CTD042	20191109	300m	-25.00333	-15.4425
CTD042	20191109	120m	-25.00333	-15.4425
CTD042	20191109	2m	-25.00333	-15.4425
CTD044	20191111	2000m	-24.83749	-21.4375
CTD044	20191111	1500m	-24.83749	-21.4375
CTD044	20191111	835m	-24.83749	-21.4375
CTD044	20191111	500m	-24.83749	-21.4375
CTD044	20191111	320m	-24.83749	-21.4375
CTD044	20191111	162m	-24.83749	-21.4375
CTD044	20191111	2m	-24.83749	-21.4375
CTD046	20191112	2000m	-25.20465	-24.735
CTD046	20191112	1500m	-25.20465	-24.735
CTD046	20191112	850m	-25.20465	-24.735
CTD046	20191112	500m	-25.20465	-24.735
CTD046	20191112	320m	-25.20465	-24.735
CTD046	20191112	158m	-25.20465	-24.735
CTD046	20191112	2m	-25.20465	-24.735
CTD048	20191113	1000m	-26.08517	-27.70883
CTD048	20191113	800m	-26.08517	-27.70883
CTD048	20191113	500m	-26.08517	-27.70883
CTD048	20191113	300m	-26.08517	-27.70883
CTD048	20191113	200m	-26.08517	-27.70883
CTD048	20191113	125m	-26.08517	-27.70883
CTD048	20191113	2m	-26.08517	-27.70883

CTD050	20191114	1000m	-27.14951	-31.21183
CTD050	20191114	800m	-27.14951	-31.21183
CTD050	20191114	500m	-27.14951	-31.21183
CTD050	20191114	300m	-27.14951	-31.21183
CTD050	20191114	200m	-27.14951	-31.21183
CTD050	20191114	125m	-27.14951	-31.21183
CTD050	20191114	2m	-27.14951	-31.21183
CTD052	20191115	1000m	-28.25201	-34.777
CTD052	20191115	900m	-28.25201	-34.777
CTD052	20191115	700m	-28.25201	-34.777
CTD052	20191115	400m	-28.25201	-34.777
CTD052	20191115	140m	-28.25201	-34.777
CTD052	20191115	85m	-28.25201	-34.777
CTD052	20191115	2m	-28.25201	-34.777
CTD054	20191117	1000m	-31.03934	-40.3555
CTD054	20191117	800m	-31.03934	-40.3555
CTD054	20191117	600m	-31.03934	-40.3555
CTD054	20191117	400m	-31.03934	-40.3555
CTD054	20191117	200m	-31.03934	-40.3555
CTD054	20191117	40m	-31.03934	-40.3555
CTD054	20191117	2m	-31.03934	-40.3555
CTD055	20191118	2000m	-35.4305	-41.90133
CTD055	20191118	1500m	-35.4305	-41.90133
CTD055	20191118	1000m	-35.4305	-41.90133
CTD055	20191118	400m	-35.4305	-41.90133
CTD055	20191118	200m	-35.4305	-41.90133
CTD055	20191118	20m	-35.4305	-41.90133
CTD055	20191118	2m	-35.4305	-41.90133

Table S2. *Summary of the core water masses of the Atlantic Ocean (adapted and modified from Emery, 2001; Emery and Meincke 1986[3,4]).*

Layer	Water mass	Properties (Temperature&Salinity)

Upper waters (0-500 m)	Atlantic Subarctic Upper Water (ASUW)	0.0-4.0 ℃, 34.0-35.0 ‰
	Western North Atlantic Central Water (WNACW)	7.0-20.0 ℃, 35.0-36.7 ‰
	Eastern North Atlantic Central Water (ENACW)	8.0-18.0 ℃, 35.2-36.7 ‰
	South Atlantic Central Water (SACW)	5.0-18.0 ℃, 34.3-35.8 ‰
Intermediate waters (500-1500 m)	Western Atlantic Subarctic Intermediate Water (WASIW)	3.0–9.0 ℃, 34.0–35.1 ‰
	Eastern Atlantic Subarctic Intermediate Water (EASIW)	3.0–9.0 ℃, 34.4–35.3 ‰
	Antarctic Intermediate Water (AAIW)	2.0-6.0 ℃, 33.8-34.8 ‰
	Mediterranean Water (MW)	2.6-11.0 ℃, 35.0-36.2 ‰
	Arctic Intermediate Water (AIW)	-1.5-3.0 ℃, 34.7-34.9 ‰
Deep and abyssal waters (1500 m-bottom)	North Atlantic Deep Water (NADW)	1.5-4.0 ℃, 34.8-35.0 ‰
	Antarctic Bottom Water (AABW)	-0.9-1.7 ℃, 34.64-34.72 ‰
	Arctic Bottom Water (ABW)	-1.8 to -0.5 ℃, 34.88-34.94 ‰

Table S3. *List of target analytes with their respective names, abbreviations, molecular formulas and standards.*

**Name**	**Abbreviation**	**Molecular formula**	**Labeled standard**	**Native standard**
Perfluorobutanoic acid	PFBA	$C_4HF_7O_2$	$^{13}C_4$-PFBA	PFBA
Perfluoropentanoic acid	PFPeA	$C_5HF_9O_2$	$^{13}C_5$-PFPeA	PFPeA
Perfluorohexanoic acid	PFHxA	$C_6HF_{11}O_2$	$^{13}C_2$-PFHxA	PFHxA
Perfluoroheptanoic acid	PFHpA	$C_7HF_{13}O_2$	$^{13}C_4$-PFHpA	PFHpA
Perfluorooctanoic acid	PFOA	$C_8HF_{15}O_2$	$^{13}C_4$-PFOA	T-PFOA
Perfluorononanoic acid	PFNA	$C_9HF_{17}O_2$	$^{13}C_5$-PFNA	PFNA
Perfluorodecanoic acid	PFDA	$C_{10}HF_{19}O_2$	$^{13}C_2$-PFDA	PFDA
Perfluoroundecanoic acid	PFUnDA	$C_{11}HF_{21}O_2$	$^{13}C_2$-PFUnDA	PFUnDA
Perfluorododecanoic acid	PFDoDA	$C_{12}HF_{22}O_2$	$^{13}C_2$-DoDA	PFDoDA

Perfluorobutanesulfonic acid	PFBS	$C_4HF_9O_3S$	$^{18}O_2$-PFHxS	L-PFHxS
Perfluorohexanesulfonic acid	PFHxS	$C_6HF_{13}O_3S$	$^{18}O_2$-PFHxS	L-PFHxS
Perfluorooctanesulfonic acid	PFOS	$C_8HF_{17}O_3S$	$^{13}C_4$-PFOS	T-PFOS

Table S4. *LC gradient program for the mobile phases A and B.*

Time [min]	Mobile Phase A %	Mobile Phase B %
0.0	90	10
0.5	90	10
8.0	20	80
8.1	0	100
11.0	0	100
11.1	90	10
13.0	90	10

Table S5. *Settings for the mass-spectrometer.*

Scan parameters		HESI source	
**Scan Type**	Full MS	**Sheath gas flow rate**	30
**Scan range**	200-1800 m/z	**Aux gas flow**	10
**Resolution**	120000	**Sweep gas flow rate**	0
**Polarity**	Negative	**Spray voltage**	3.70 kV
		**Capillary temp.**	350 °C
		**S-lens RF level**	55
		**Aux gas heater temp.**	350 °C

Table S6. *Results of the spike recovery test for PFCAs C5-C12 and PFSAs C4, C6 and C8 which were spiked with 50 pg and 100 ng of the respective compound.*

	Spiked 50 pg	Spiked 100 ng	Measured concentration pg, ng		Recovery	
**PFPeA**	51.1	99.4	45.4	97.0	89%	98%

**PFHxA**	51.2	99.6	49	102.3	96%	103%
**PFHpA**	51.1	99.4	54	104.5	106%	105%
**PFOA**	40.2	78.2	39	76.8	97%	98%
**PFNA**	52.6	102.3	48	104.3	91%	102%
**PFDA**	51.7	100.5	47	88.6	91%	88%
**PFUnDA**	50.8	98.8	36	80.5	71%	81%
**PFDoDA**	51.8	100.6	27	67.0	52%	67%
**PFBS**	50.8	98.8	46	79.7	90%	81%
**PFHxS**	51.3	99.7	54	112.8	105%	113%
**PFOS**	28.0	54.3	23	54.1	82%	100%

Table S7. *Recovery range and median (%) for the internal standards of the target compounds.*

IS	Range	Median
**MPFHxA**	6-97%	37%
**MPFHpA**	10-97%	46%
**MPFOA**	24-110%	85%
**MPFNA**	14-277%	88%
**MPFDA**	17-316%	85%
**MPFUnDA**	21-275%	83%
**MPFDoDA**	34-372%	95%
**MPFHxS**	16-153%	77%
**MPFOS**	23-102%	79%

Table S8. *Preparation of the CTD samples in cartridge (batch 1-3) and elution batches (A-E). With *5 to 5000m; **2 or 5 m.*

CTD No.	Deeper water*	Surface**	Field blank	Elution batch

**CTD002**	Batch 3	Batch 3		A
**CTD004**	Batch 3	Batch 3		B
**CTD006**	Batch 3	Batch 3		B
**CTD008**	Batch 3	Batch 3		C
**CTD011**	Batch 3	Batch 3		C
**CTD013**	Batch 3	Batch 3		D
**CTD014**	Batch 3	Batch 3		A
**CTD016**	Batch 3	Batch 3		B
**CTD018**	Batch 2	Batch 3		B
**CTD020**	Batch 2	Batch 3	Batch 1	C
**CTD021**	Batch 2	Batch 2		D
**CTD023**	Batch 2	Batch 2	Batch 1	C
**CTD025**	Batch 2	Batch 2		D
**CTD027**	Batch 2	Batch 2	Batch 1	D
**CTD029**	Batch 2	Batch 2		D
**CTD031**	Batch 2	Batch 2	Batch 1	D
**CTD033**	Batch 2	Batch 2		C
**CTD034**	Batch 2	Batch 2	Batch 1	E
**CTD036**	Batch 2	Batch 2		B
**CTD038**	Batch 2	Batch 2	Batch 1	B
**CTD040**	Batch 2	Batch 2	Batch 1	C
**CTD042**	Batch 2	Batch 2	Batch 1	D
**CTD044**	Batch 2	Batch 2	Batch 1	A
**CTD046**	Batch 2	Batch 2	Batch 1	D
**CTD048**	Batch 2	Batch 2	Batch 1	B
**CTD050**	Batch 2	Batch 2	Batch 1	C
**CTD052**	Batch 2	Batch 2	Batch 1	C
**CTD054**	Batch 2	Batch 2	Batch 1	A
**CTD055**	Batch 2	Batch 2	Batch 2	B

Table S9. *Detection and quantification limits [pg L^{-1}] for target compounds in the different elution batches A-D.*

	**Batch A**		**Batch B**		**Batch C**		**Batch D**	
	**MDL**	**MQL**	**MDL**	**MQL**	**MDL**	**MQL**	**MDL**	**MQL**
**PFHxA**	4.39	9.85	2.04	6.80	5.77	14.20	3.56	7.82
**PFHpA**	1.90	4.88	0.99	2.22	1.84	4.68	1.30	3.17
***br*-PFOA**	12.07	32.25	6.59	16.76	5.95	13.26	6.26	15.91
**PFNA**	1.35	3.46	1.27	3.32	2.84	7.68	1.84	4.82
**PFDA**	1.24	3.28	1.40	3.39	1.88	4.94	1.74	4.61
**PFUnDA**	0.45	0.95	0.61	1.38	0.82	2.14	0.46	1.11
**PFDoDA**	0.30	0.51	0.30	0.51	0.75	2.10	0.30	0.51
**PFHxS**	1.84	4.51	3.08	8.49	0.88	1.58	1.84	4.51
***l*-PFOS**	5.31	14.65	7.58	21.77	2.46	6.08	5.04	13.40
***br*-PFOS**	2.53	7.16	6.43	19.03	2.00	5.17	5.60	15.21

Table S10. *Detection and quantification limits [pg L^{-1}] for l-PFOA in the three cartridge batches.*

	**Batch 1**	**Batch 2**	**Batch 3**
**MDL**	27.85	4.23	27.85
**MQL**	92.83	14.10	92.83

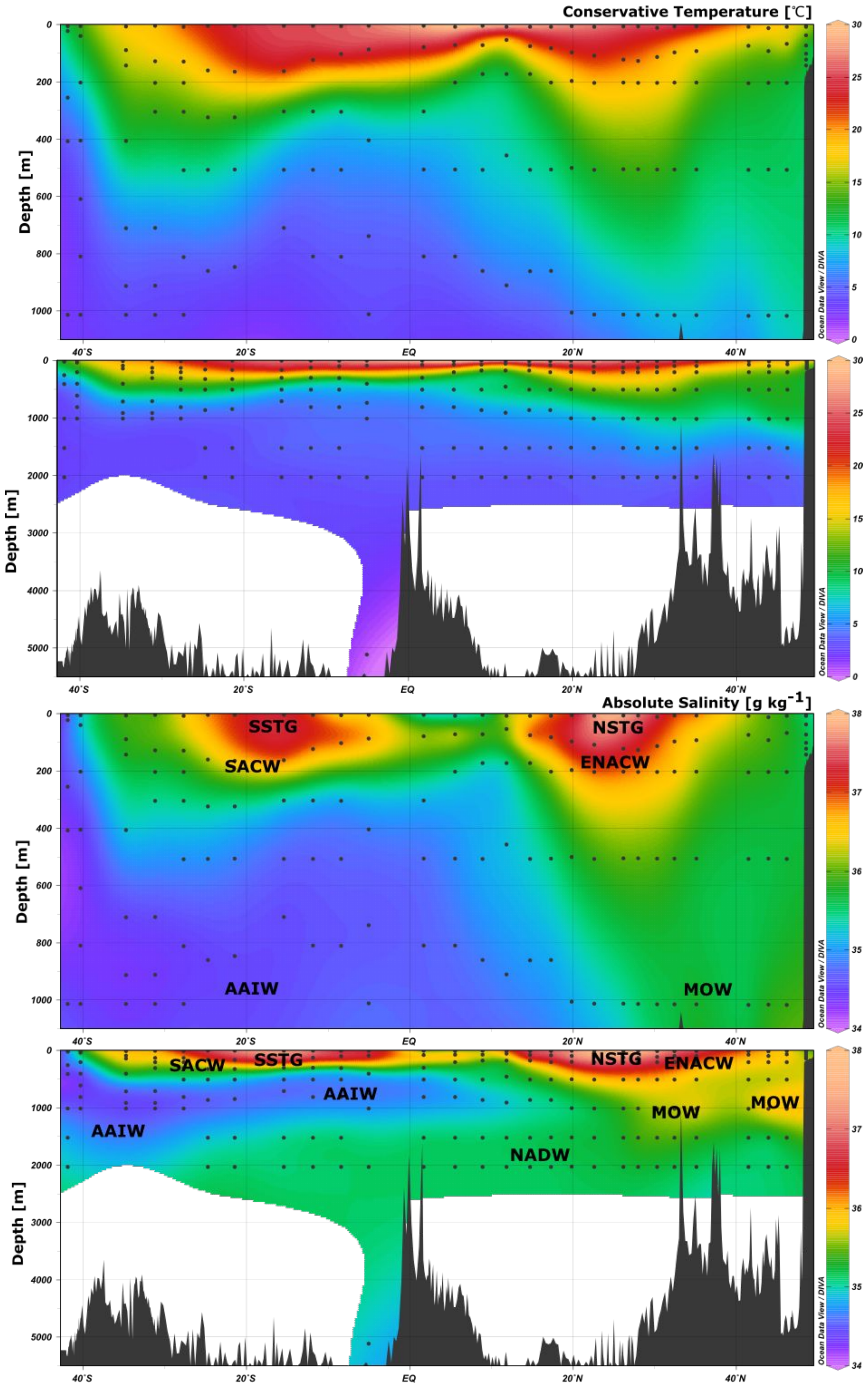

Figure S1. *Temperature and salinity plots for depths from 0-1000m (top) and 0-5000 m (bottom). The salinity plot includes the identified water masses.*

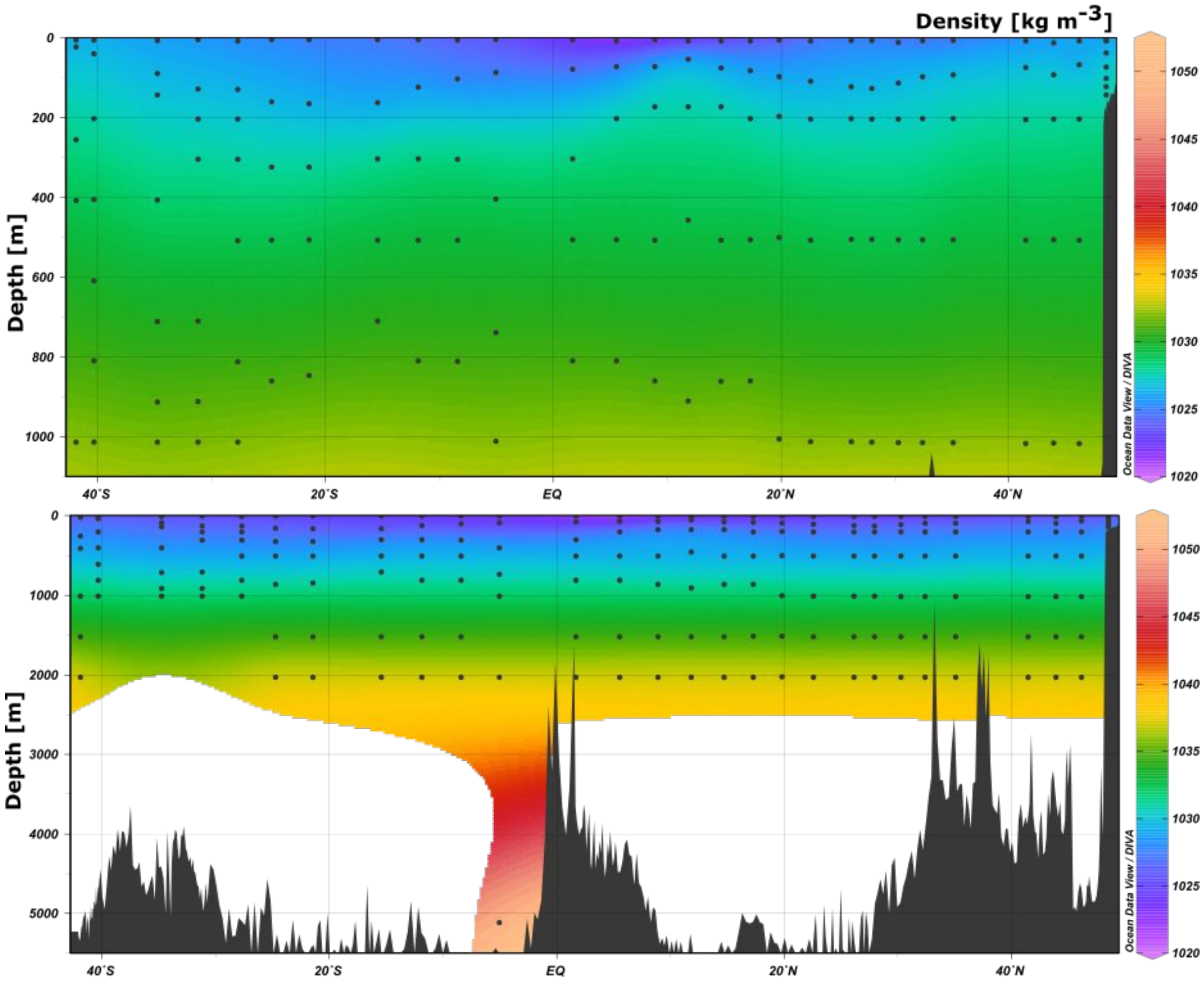

Figure S2. *Density plot for depths from 0-1000 m (top) and 0-5000m (bottom). The black dots represent the sampling points.*

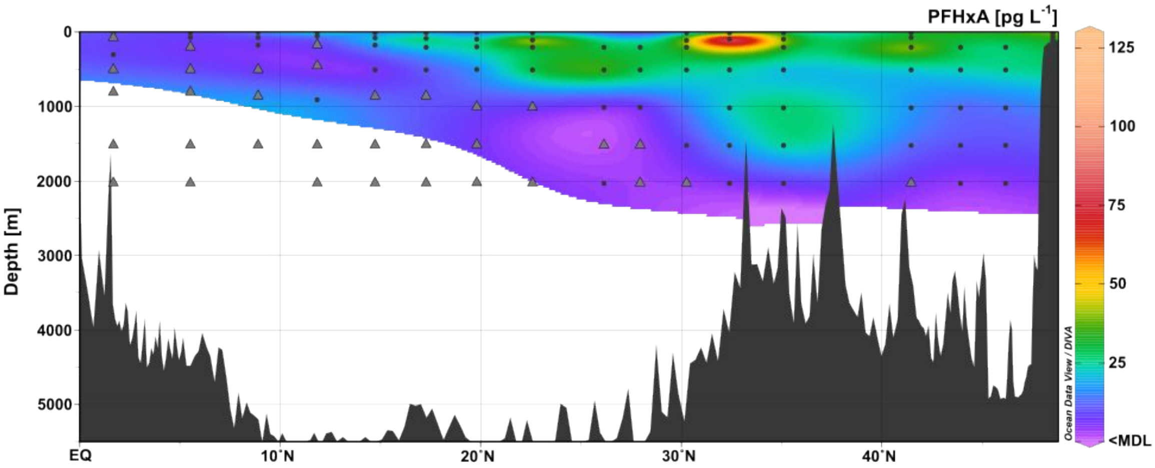

Figure S3. *Distribution of PFHxA in the vertical water column in the Northern Hemisphere. Black dots represent the sampling stations, grey triangles are non-detects (<MDL).*

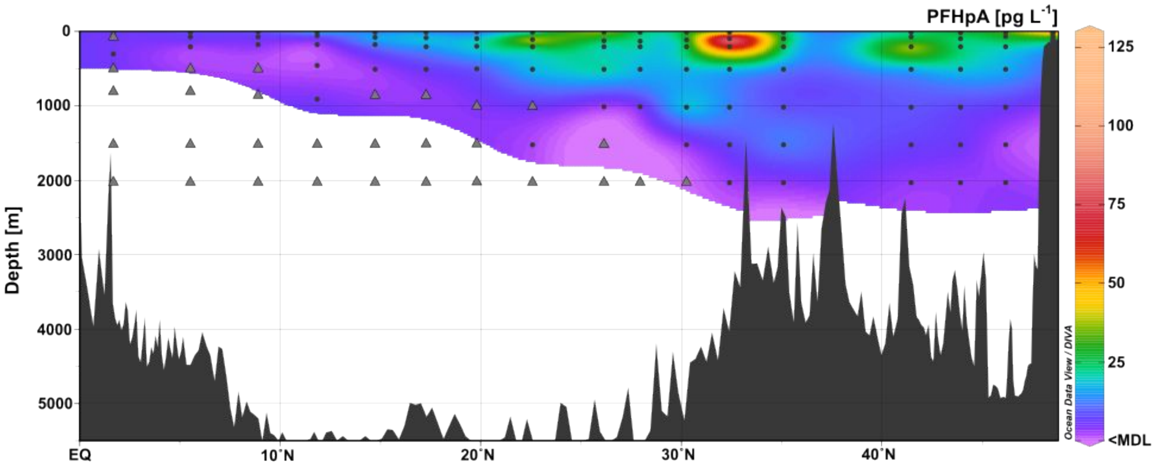

Figure S4. *Distribution of PFHpA in the vertical water column in the Northern Hemisphere. Black dots represent the sampling stations, grey triangles are non-detects (<MDL).*

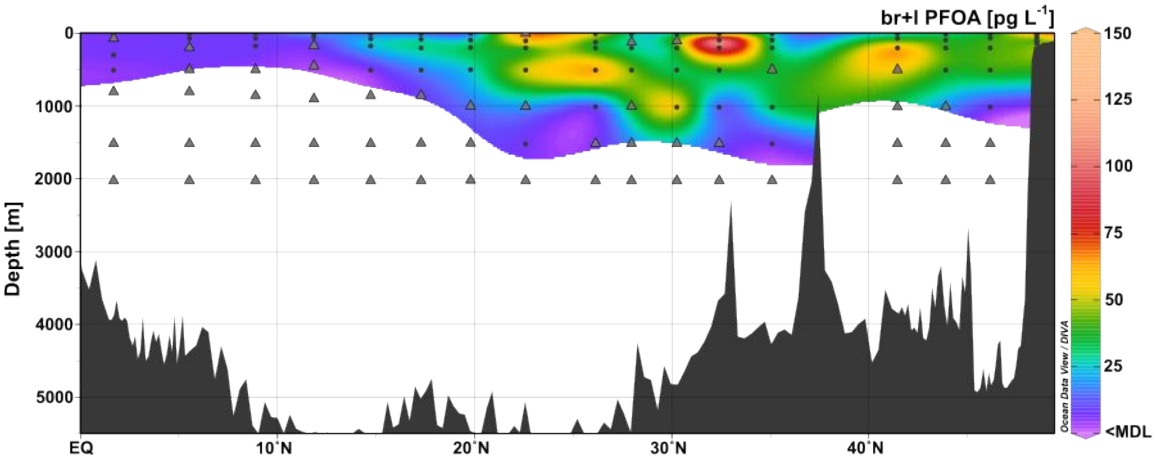

Figure S5. *Distribution of branched and linear PFOA in the vertical water column in the Northern Hemisphere. Black dots represent the sampling stations, grey triangles are non-detects (<MDL).*

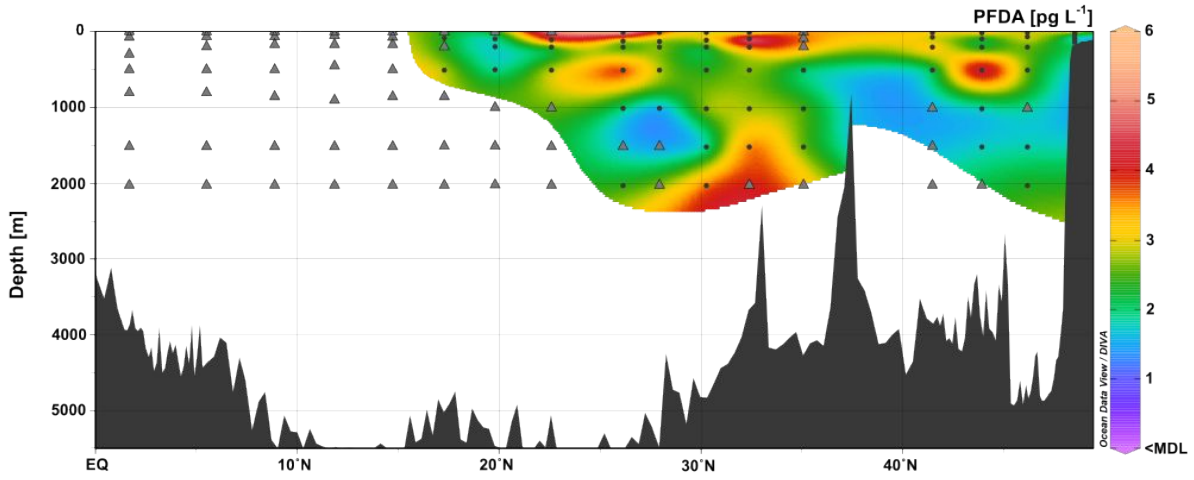

Figure S6. *Distribution of PFDA in the vertical water column in the Northern Hemisphere. Black dots represent the sampling stations, grey triangles are non-detects (<MDL).*

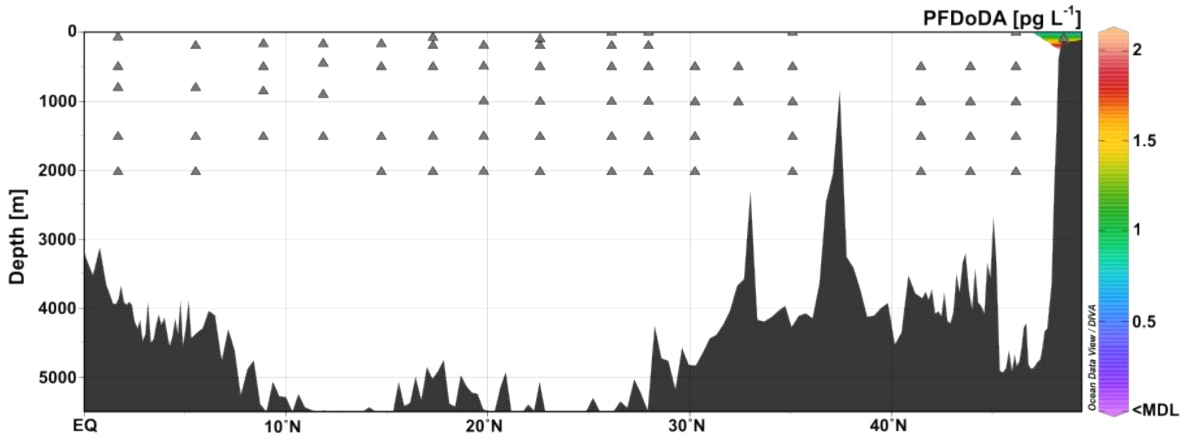

Figure S7. *Distribution of PFDoDA in the vertical water column in the Northern Hemisphere. Black dots represent the sampling stations, grey triangles are non-detects (<MDL)*

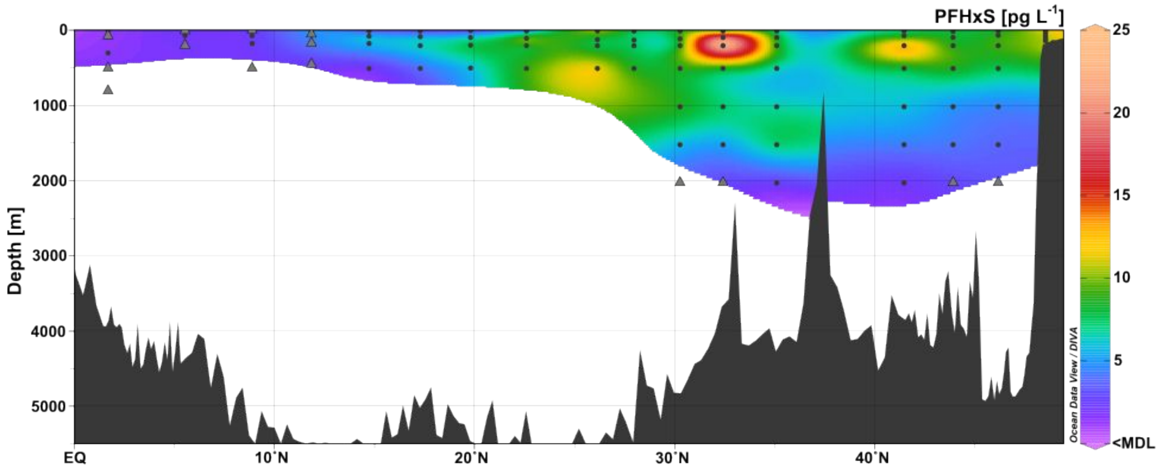

Figure S8. *Distribution of PFHxS in the vertical water column in the Northern Hemisphere. Black dots represent the sampling stations, grey triangles are non-detects (<MDL).*

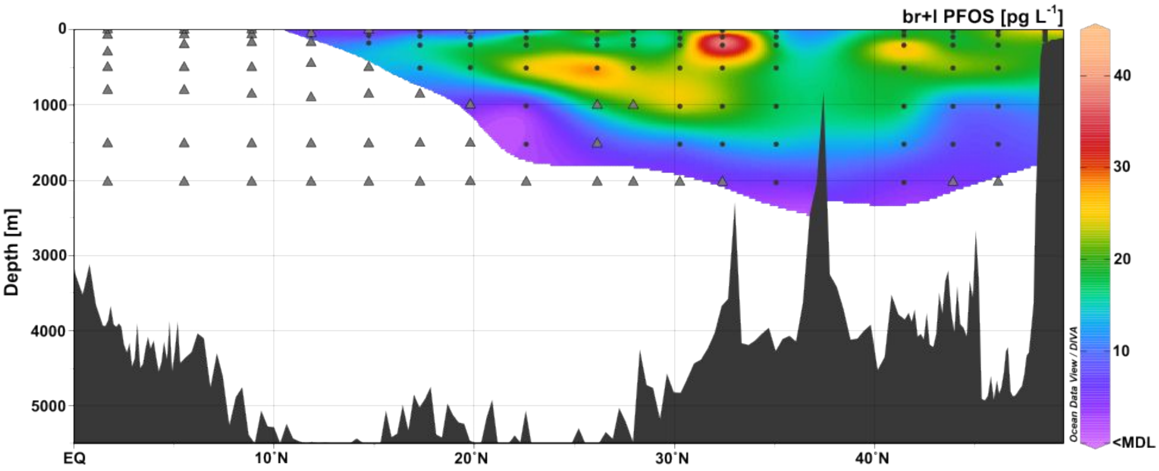

Figure S9. *Distribution of branched and linear PFOS in the vertical water column in the Northern Hemisphere. Black dots represent the sampling stations, grey triangles are non-detects (<MDL).*

References

(1) Frazão, H. C.; Waniek, J. J. Mediterranean Water Properties at the Eastern Limit of the North Atlantic Subtropical Gyre since 1981. *Oceans* **2021**, *2* (1), 266–280. https://doi.org/10.3390/oceans2010016.

(2) Löfstedt Gilljam, J.; Leonel, J.; Cousins, I. T.; Benskin, J. P. Is Ongoing Sulfluramid Use in South America a Significant Source of Perfluorooctanesulfonate (PFOS)? Production Inventories, Environmental Fate, and Local Occurrence. *Environ. Sci. Technol.* **2016**, *50* (2), 653–659. https://doi.org/10.1021/acs.est.5b04544.

(3) Emery, W. J. Water Types And Water Masses. In *Encyclopedia of Ocean Sciences*; Steele, J. H., Ed.; Academic Press: Oxford, 2001; pp 3179–3187. https://doi.org/10.1006/rwos.2001.0108.

(4) Emery, W.J.; Meincke J. Global Water Masses : Summary and Review. *Oceanol. Acta* **1986**, 9 (4), 383-391.
